# 

**Published:** 1876-01

**Authors:** 


[illegible]. 23. JANUARY, 1876. No. 1.

TWENTY-THIRD YEAR.

### HALL'S Journal of Health.

PUBLISHED AT 137 EIGHTH ST., NEW YORK.

Four doors East of 756 Broadway.

COPYRIGHT SECURED 1876.

**Agents for the Trade:**

AMERICAN NEWS COMPANY, 121 NASSAU STREET.
NEW YORK NEWS COMPANY, 18 BEEKMAN STREET.

**Terms, Two Dollars a Year.**

**SINGLE NUMBER, 20 CENTS.**

